# No Sex Differences in Use of Dopaminergic Medication in Early Parkinson Disease in the US and Canada - Baseline Findings of a Multicenter Trial

**DOI:** 10.1371/journal.pone.0112287

**Published:** 2014-12-08

**Authors:** Chizoba C. Umeh, Adriana Pérez, Erika F. Augustine, Rohit Dhall, Richard B. Dewey, Zoltan Mari, David K. Simon, Anne-Marie A. Wills, Chadwick W. Christine, Jay S. Schneider, Oksana Suchowersky

**Affiliations:** 1 Department of Neurology and Harvard Medical School, Brigham and Women's Hospital, Boston, Massachusetts, United States of America; 2 Department of Biostatistics, The University of Texas School of Public Health, Austin, Texas, United States of America; 3 Department of Neurology, University of Rochester Medical Center, Rochester, New York, United States of America; 4 Department of Neurology, Barrow Neurological Institute, Phoenix, Arizona, United States of America; 5 Department of Neurology and Neurotherapeutics, University of Texas Southwestern Medical Center, Dallas, Texas, United States of America; 6 Department of Neurology, Johns Hopkins University, Baltimore, Maryland, United States of America; 7 Department of Neurology, Beth Israel Deaconess Medical Center and Harvard Medical School, Boston, Massachusetts, United States of America; 8 Department of Neurology, Massachusetts General Hospital, Boston, Massachusetts, United States of America; 9 Department of Neurology, University of California San Francisco, San Francisco, California, United States of America; 10 Department of Pathology, Anatomy and Cell Biology, Thomas Jefferson University, Philadelphia, Pennsylvania, United States of America; 11 Departments of Medicine (Neurology), Medical Genetics and Pediatrics, University of Alberta, Edmonton, Alberta, Canada; University of Ulm, Germany

## Abstract

**Background:**

Sex differences in Parkinson disease clinical features have been reported, but few studies have examined sex influences on use of dopaminergic medication in early Parkinson disease. The objective of this study was to test if there are differences in the type of dopaminergic medication used and levodopa equivalent daily dose between men and women with early Parkinson disease enrolled in a large multicenter study of Creatine as a potential disease modifying therapy – the National Institute of Neurological Disorders and Stroke Exploratory Trials in Parkinson Disease Long-Term Study-1.

**Methods:**

Baseline data of 1,741 participants from 45 participating sites were analyzed. Participants from the United States and Canada were enrolled within five years of Parkinson Disease diagnosis. Two outcome variables were studied: type of dopaminergic medication used and levodopa equivalent daily dose at baseline in the Long-Term Study-1. Chi-square statistic and linear regression models were used for statistical analysis.

**Results:**

There were no statistically significant differences in the frequency of use of different types of dopaminergic medications at baseline between men and women with Parkinson Disease. A small but statistically significant difference was observed in the median unadjusted levodopa equivalent daily dose at baseline between women (300 mg) and men (325 mg), but this was not observed after controlling for disease duration (years since Parkinson disease diagnosis), disease severity (Unified Parkinson's Disease Rating Scale Motor and Activities of Daily Living Scores), and body weight.

**Conclusions:**

In this large multicenter study, we did not observe sex differences in the type and dose of dopaminergic medications used in early Parkinson Disease. Further research is needed to evaluate the influence of male or female sex on use of dopaminergic medication in mid- and late-stage Parkinson Disease.

## Introduction

Sex differences in medical treatment have been reported in the pain and cardiovascular medicine literature, with men receiving more aggressive treatment for these conditions. [1], [2] When assessing sex differences in stroke- a prevalent neurological condition- women are less likely than men to receive thrombolytic treatment with alteplase and are reported to have poorer stroke-related outcomes including greater disability and lower quality of life. [3] Disparities in treatment based on sex can have a significant impact on morbidity, mortality, and quality of life.

Sex differences in the clinical characteristics of Parkinson Disease (PD) have been frequently reported, however, little is known about the influence of male or female sex on treatment for this common condition. The initial presenting sign, severity of parkinsonian symptoms, and/or development of dyskinesia are important considerations in clinical practice regarding choice of dopaminergic medication in PD. In a study of 1,264 PD patients with similar disease duration, men were found to have more severe rigidity while women had more severe dyskinesias with treatment. [4] Another study showed that women were more likely than men to have tremor as an initial presenting sign. [5] These sex differences in clinical characteristics could influence the type and dose of dopaminergic medications used in early PD. With the exception of a few studies, the impact of male or female sex on dopaminergic medication use in PD has remained largely unexplored.

In one study of PD subjects with disease duration of at least 5 years, there was a male predominance in the high dose levodopa group. [6] In another study by Lyons et al., men with disease duration of greater than 5 years were taking significantly higher daily doses of levodopa compared to women of similar disease duration, but this sex difference was not present in subjects with disease duration less than 5 years. [7] Despite these previous studies comparing dopaminergic medication use in men versus women with PD, there is no clear consensus on whether choice of dopaminergic medication is influenced by sex in the early stages of PD.

The type and dose of dopaminergic medication can affect the rate of development of motor complications. For example, a previous study showed that patients randomized to levodopa developed motor fluctuations at higher rates than those first treated with a dopamine agonist. [8] Moreover, the levodopa daily dose used in early PD may impact the development of motor complications. Indeed, participants in the Earlier versus Later Levodopa Therapy in Parkinson Disease study receiving the highest levodopa doses had a higher risk of developing dyskinesias. [9] To our knowledge, there are no prior studies examining the specific types of dopaminergic medications used between men and women with early PD. Knowledge of differential exposure to dopaminergic medications according to sex could inform future clinical management. The objective of this secondary data analysis was to examine sex influences on treatment by comparing the type of dopaminergic medication and the levodopa equivalent daily dose (LEDD) between men and women using baseline data from early treated PD patients who were enrolled in the National Institute of Neurological Disorders and Stroke Exploratory Trials in Parkinson Disease (NINDS NET-PD) Long-Term Study-1 (LS-1). [10]

## Methods

The NINDS NET-PD LS-1 is a randomized, multicenter, double-blind study of 10 grams of oral creatine/day versus matching placebo in adults with early treated PD enrolled within five years of PD diagnosis (clinicaltrials.gov identifier# NCT00449865). [10] The LS-1 study enrolled 1,741 participants from 45 sites in the United States and Canada between March 13, 2007 and May 28, 2010. The study protocol was approved by the NINDS NET-PD LS-1 Steering Committee and Research Subjects Review Board of the University of Rochester. In addition, an institutional review board (IRB) from each participating site approved the study protocol and written informed consent was obtained from each study participant. [10] Participating sites/IRBs included: University of Alabama-Birmingham, University of South Florida, University of Southern California, Emory University School of Medicine, Oregon Health & Science University, University of Colorado, Johns Hopkins University, University of Texas Southwestern Medical Center, University of California San Francisco, University of Florida, Duke University, Louisiana State University Health Science Center-Shreveport, Michigan State University, Rush University Medical Center, University of Calgary, University of Pennsylvania, Beth Israel Deaconess Medical Center, Southern Illinois University, University of Michigan, Brigham and Women's Hospital, University of Miami, Medical University of South Carolina, Pacific Health Research and Education Institute, University of Alberta, Washington University, University of Maryland School of Medicine, University of Vermont, Northwestern University, University of Kansas Medical Center, University of Kentucky, Dartmouth Hitchcock Medical Center, SUNY Downstate Medical Center, Thomas Jefferson University, Baylor College of Medicine, Georgia Health Sciences University, Institute for Neurodegenerative Disorders-New Haven, The Parkinson's & Movement Disorder Institute-Fountain Valley, University of Virginia, Vanderbilt University Medical Center, Barrow Neurological Institute, UMDNJ Robert Wood Johnson Medical School, Malcolm Randall VA Medical Center, University of Florida-Jacksonville, Indiana University School of Medicine, and North Shore University Health System Research Institute. [10]

We considered the following hypotheses: 1.) there are sex differences in the proportions of type of dopaminergic medication at baseline and 2.) there are sex differences in the means of the LEDD at baseline. Two outcome variables were considered for this study: type of dopaminergic medication and LEDD at baseline for NINDS NET-PD LS-1.

Pre-planned adjusting factors (for LEDD) at baseline included: disease duration (years since PD diagnosis), disease severity (Unified Parkinson's Disease Rating Scale (UPDRS) Motor + Activities of Daily Living (ADL) Scores), and body weight. Originally, six categories were identified in the type of dopaminergic medication used at enrollment of LS-1 participants: 1. levodopa alone; 2. levodopa + other medication (different from dopamine agonist), 3. dopamine agonist alone, 4. dopamine agonist + other medication (different from levodopa), 5. dopamine agonist + levodopa, and 6. other medication (rasagiline or trihexyphenidyl). These six categories were used when testing the hypothesis of differences between men and women in the type of dopaminergic medication taken at baseline. For testing the hypothesis of differences in the LEDD between men and women, we collapsed PD treatment into three main groups similar to the previously published baseline LS-1 results paper [10]: levodopa alone, dopamine agonist alone, and more than one PD medication.

## Statistical Methods

Descriptive statistics of demographic characteristics, age at symptom onset and age at PD diagnosis, as well as motor symptoms were estimated as means or proportions. The comparison of age and motor symptoms between men and women were estimated using t-statistics or the global O'brien non-parametric rank sum test. The proportion of type of dopaminergic medication between men and women was compared using a chi-square statistic. The LEDD was computed using the method described by Tomlinson [11] with a minor modification: LEDD calculation for Stalevo was Levodopa dose ×1.33. Following the recommendation of the Box-Cox transformation method, LEDD was transformed using the natural logarithm (Ln) to improve normality. [12] The median and the interquartile range (IQR) of the LEDD for men and women are reported by type of dopaminergic medication at baseline. Summary statistics of the LEDD are presented as well as for the Ln LEDD at baseline in the NINDS NET-PD LS-1 to facilitate the clinical understanding of LEDD in the original scale and the transformed variable. The median LEDD between men and women was compared using a two-tailed median two sample test. Using a linear regression model, we explored the differences in the natural logarithm of LEDD between men and women adjusted for body weight, disease duration (years since PD diagnosis), and disease severity (UPDRS motor + ADL). Ten participants with missing LEDD, UPDRS motor or ADL scores were excluded from the LEDD analysis. A type I error level value of 0.05 or less was established *a priori* as significant.

The natural logarithm of LEDD was adjusted on two sets of pre-planned covariates. The first model included sex, years since PD diagnosis, body weight and UPDRS motor + ADL scores. The second model included sex, years since PD diagnosis, and body weight. The UPDRS motor + ADL scores were divided by 10 to be able to visualize the regression coefficient estimates with 4 decimal places. Partial F-test statistics were used to compare model 1 and 2.

## Results

On average, the LS-1 baseline cohort had mild motor impairment and a mean age of 61.8 years. [10] There were a greater proportion of men (64.5%) than women enrolled in the study. [10] Men and women in LS-1 had overall similar demographic and clinical characteristics at baseline. The percentage of women with a mean age above 50 was 86.6% compared to 90% of men. The largest ethnic category of participants was non-hispanic whites (90.1% of men and 90.5% of women). There were similarities in educational background with 79.2% of women attaining some college education or higher educational status compared to 84.2% of men.

We did not observe statistically significant differences in the mean age at first PD symptom onset between women (58.1, standard deviation(SD) = 10.4) and men (58.7, SD = 9.6), t-statistic for unequal variances = −1.27, df = 1186.3, p-value = 0.20; or in the mean age at PD diagnosis in women (59.8, SD = 10.1) and men (60.5, SD = 9.4), t-statistic for unequal variances  = −1.37, df = 1191.7, p-value = 0.17. There were no statistically significant differences between men and women with regard to motor signs present at baseline: resting tremor (women: 82.8%, men: 78%), rigidity (women: 86.5%, men: 87.5%), bradykinesia (women: 91.9%, men: 91.6%), postural instability (women: 20.7%, men: 18.5%) and other motor symptoms (women: 17.9%, men = 16.9%), global O'Brien nonparametric rank sum test, F = 3.3, df = 1675, p-value = 0.0693. The mean (SD) UPDRS motor score for women was 17 (8.6) compared to 18.2 (8.2) for men (t-statistic for equal variances = 2.97, df = 1731, p-value = 0.003).

Table 1 shows the frequency distribution of dopaminergic medication at baseline in men and women. Among women, almost 28% were taking levodopa alone, 27% were taking dopamine agonist alone, 19% were taking the combination of both dopamine agonist and levodopa, 17% were taking dopamine agonist and other medication different from levodopa, 8% were taking levodopa and other medication different from dopamine agonist and 0.5% were taking other medications different from levodopa or dopamine agonist. Similar percentages were observed among men and we did not observe statistically significant differences in the proportions of PD dopaminergic medication used at baseline between men and women (Chi-square statistic = 3.61, df = 5, p-value = 0.61).

**Table 1:** Type of PD dopaminergic medication use at baseline: descriptive statistics and test.

Type of PD Dopaminergic medication	Female		Male	
	n	%	n	%
Levodopa only	173	28.0	332	29.6
Levodopa + Other* (different from dopamine agonist)	52	8.4	114	10.2
Dopamine agonist alone	167	27.0	296	26.4
Dopamine agonist + Other* (different from Levodopa)	104	16.8	178	15.9
Dopamine agonist + Levodopa	119	19.3	201	17.9
Other* (different from Levodopa or dopamine agonist)	3	0.5	2	0.2

Chi-square statistic = 3.61, degrees of freedom = 5, p-value = 0.6054.

*Other medications included rasagiline or trihexyphenidyl.

**Figure 1:**
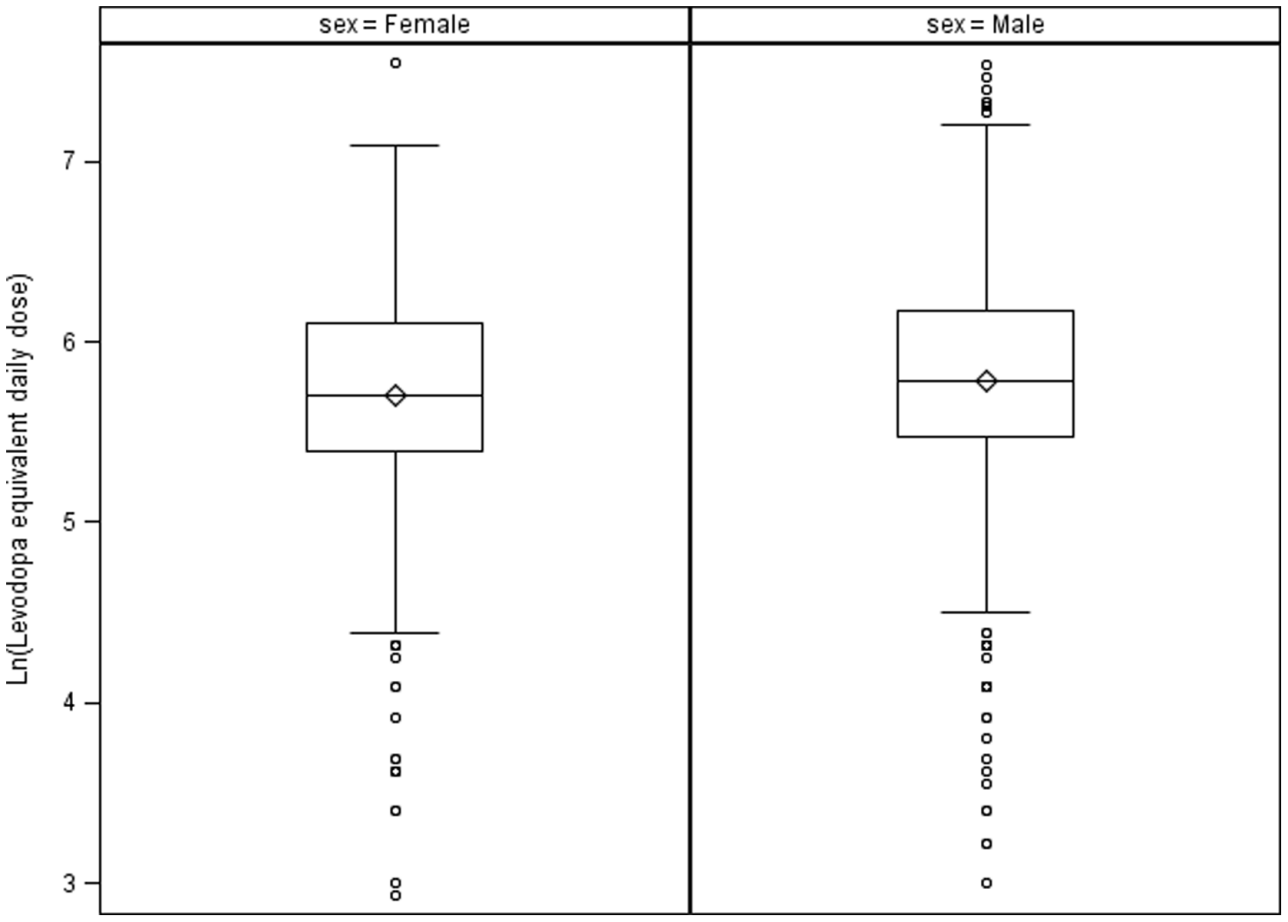
Box Plot: Natural logarithm of levodopa equivalent daily dose displayed by sex.

Table 2 shows the sample size, the median, and the interquartile range (IQR) of the LEDD by sex and type of dopaminergic medication. The mean and standard deviation of the Ln LEDD is also shown in Table 2. Fig. 1 displays the box plot of Ln LEDD by sex. The unadjusted median LEDD at baseline was 300 mg (IQR = 230) for women versus 325 mg for men (IQR = 240 mg) (data not shown). We detected a significant difference in the median unadjusted LEDD between men and women (median two sample test = 282.46, two sided p-value = 0.0066). However, after adjusting the Ln LEDD for body weight, disease duration (years since PD diagnosis), and disease severity (UPDRS motor +ADL), this difference was no longer significant (Table 3).

**Table 2:** Summary statistics of the levodopa equivalent daily dose (LEDD) and natural logarithm LEDD (Ln LEDD) at baseline.

Sex	Type of dopaminergic medication at baseline	n	LEDD (mg)		Ln LEDD	
			Median	Interquartile Range	Mean	SD
Female	Levodopa alone	172	300	100	5.85	0.43
	Dopamine agonist alone	165	180	90	5.13	0.64
	More than one PD medication	277	400	275	5.96	0.53
Male	Levodopa alone	330	399	250	5.98	0.44
	Dopamine agonist alone	294	180	150	5.22	0.61
	More than one PD medication	493	400	255.5	6.00	0.52

**Table 3:** Ln (Levodopa equivalent daily dose) regressed on sex, years since PD diagnosis, body weight, and UPDRS motor +ADL Scores.

	Model 1			Model 2		
Effect	Parameter estimate	Standard error	p-value	Parameter estimate	Standard error	p-value
Intercept	5.437	0.083	<.0001	5.437	0.077	<.0001
Male	0.028	0.036	0.439	0.028	0.036	0.437
Years since PD diagnosis	0.027	0.014	0.053	0.027	0.014	0.050
Body Weight (kg)	0.003	0.001	0.001	0.003	0.001	0.001
UPDRS motor +ADL Score*	0.0003	0.014	0.985			

*UPDRS motor +ADL scores were divided by 10 to visualize the regression coefficient.

Model 1 vs Model 2, Partial F test (1,1723) = 0.00033, p-value = 0.986, Model 2 is preferred.

## Discussion

While sex differences have been shown in treatment and clinical outcomes in cardiovascular disease, pain and stroke [1], [2], [3], there has been little research on the impact of sex on treatment in other prevalent conditions including PD. Whether male or female sex impacts the type and dose of dopaminergic medication used in PD is poorly understood. Sex differences in the clinical characteristics described in the Parkinson's literature including initial presenting sign, severity of motor signs and/or development of dyskinesias [4], [5] are factors that may influence prescriber choice of dopaminergic medication in early PD. Improved understanding of sex influences on dopaminergic medication use can improve our knowledge of current treatment practices and implications for patient outcomes.

Nyholm et al [6] reported that men used larger levodopa doses than women. This study was based on estimated levodopa daily doses of subjects selected from a national drug registry without a confirmed PD diagnosis, and without adjusting for PD disease duration or body weight. [6] In their subsequent retrospective chart review of 47 PD patients with a disease duration of at least 5 years, there was a male predominance in the high-dose levodopa group. [6] In another study, Lyons et al. reviewed database entries of 630 patients from a single center's Parkinson's disease registry and examined the relationship between sex and total daily levodopa dosage using Spearman rank order correlations. [7] They reported higher levodopa use in men versus women with PD disease duration greater than 5 years; however, this difference was not present in subjects with disease duration less than 5 years. [7] Using the large NINDS NET-PD LS-1 study, we did not find differences in the use of different types of dopaminergic medications between men and women with early PD. In addition, there were no significant differences in LEDD between men and women after controlling for body weight, disease duration and disease severity.

While we found a significant difference in the unadjusted LEDD between men and women (unadjusted median LEDD at baseline was 300 mg for women versus 325 mg for men, two sided p-value = 0.0066), this difference was no longer significant after adjusting for several important variables. Compared to our analysis, the previous studies by Lyons and Nyholm et al. did not adjust for potentially confounding factors to levodopa dose including body weight. In an analysis of levodopa treatment in the DATATOP study which accounted for body weight, women were reported to be treated with higher levodopa doses in mg/kg compared to men. [13], [14] Weight differences can influence the pharmacokinetics of levodopa. [15] Zappia et al. showed that the plasma levodopa area under the curve was significantly and inversely correlated with body weight in PD patients. [15] The impact of body weight on levodopa usage by sex is unclear as most specialists do not prescribe based on body weight; however, previous studies suggest that weight differences may influence sex differences in treatment response (i.e. increased prevalence of dyskinesia in women). [15] This observation has been previously confirmed in a study comparing levodopa-carbidopa with levodopa-carbidopa-entacapone where low body weight and female sex were both associated with an increased risk of dyskinesia. [16] Future studies examining sex influences on the use of dopaminergic medications in PD should take into consideration the potential impact of body weight on treatment dose.

We did not observe sex differences in the use of dopaminergic medications in early PD subjects in the LS-1 study. Our analysis was based on baseline data from a clinical trial of 1,741 PD participants from 45 sites in the U.S. and Canada and examined the relationship between sex and LEDD using a linear regression method with adjustment for important confounding variables. We used levodopa equivalent daily dose in our analysis instead of levodopa dose alone, which allowed for a more comprehensive assessment of dopaminergic medication use in these early PD participants.

The role of prescriber choice of dopaminergic medication based on the sex of the patient is unclear and was not a specific focus of our study. Generally, it is assumed that physicians, including movement disorder specialists prescribe dopaminergic medications based on PD symptoms, disease severity, and medication side effects. Sex differences in clinical characteristics at first presentation and in the development of dyskinesia play an important role in prescriber treatment choice; however, men and women in this study had overall similar clinical characteristics at baseline including motor PD signs and prevalence of dyskinesia.

Menopausal status and estrogen replacement therapy in women may influence the severity of PD [14], [17] and thus may influence dopaminergic medication use. There are conflicting results on the impact of estrogen use on PD symptom severity, with some studies showing that estrogen use may be associated with improved motor symptoms while other studies report worsened parkinsonism with estrogen therapy. [18], [19], [20] NET-PD LS-1 did not systematically collect data on menopausal status, estrogen therapy use or hormone replacement therapies. Future studies examining sex differences in PD should consider the potential impact of menopausal status and estrogen therapy on dopaminergic medication use.

It has been reported previously that socioeconomic status influences level of utilization of medical treatment. [21] While there is a potential role of socioeconomic status (SES) on use of dopaminergic medication in PD, we observed similarities between men and women in educational level, an important component of SES. Further research is warranted to clarify the impact of socioeconomic measures on dopaminergic medication use in early PD.

Our study findings are relevant to clinical care as the type and dose of dopaminergic medication have long-term implications for motor function and quality of life. The large sample size and broad recruitment suggests that our results may be generalizable to the early PD population in North America with similar demographic characteristics as participants in LS-1; however, these results are not generalizable to all PD treatment centers. We did not observe significant differences between men and women in the type and dose of dopaminergic medication in participants with early PD in the LS-1 study. Further research is needed to understand sex influences on dopaminergic medication use in more advanced PD and the potential impact on clinical outcomes.
